# A randomized, embedded, pragmatic, Bayesian clinical trial examining clinical decision support for high flow nasal cannula management in children with bronchiolitis: design and statistical analysis plan

**DOI:** 10.1186/s13063-024-08327-y

**Published:** 2024-07-16

**Authors:** Christopher M. Horvat, Srinivasan Suresh, Nathan James, Rajesh K. Aneja, Alicia K. Au, Scott Berry, Arthur Blumer, Kelly Bricker, Robert S. B. Clark, Heidilyn Dolinich, Sheila Hahner, Christina Jockel, Jordan Kalivoda, India Loar, Denee Marasco, Adrienne Marcinick, Oscar Marroquin, Jonathan O’brien, Jonathan Pelletier, Sriram Ramgopal, Shekhar Venkataraman, Derek C. Angus, Gabriella Butler

**Affiliations:** 1 UPMC Children’s Hospital of Pittsburgh, Pittsburgh, PA USA; 2 Division of Health Informatics, Department of Pediatrics, University of Pittsburgh School of Medicine, Pittsburgh, PA USA; 3 Department of Critical Care Medicine, University of Pittsburgh School of Medicine, Pittsburgh, PA USA; 4 UPMC, Pittsburgh, PA USA; 5 Berry Statistical Consultants, Austin, TX USA; 6 Division of Pediatric Critical Care Medicine, Department of Pediatrics, Akron Children’s Hospital, Akron, OH USA; 7 Division of Emergency Medicine, Department of Pediatrics, Ann & Robert H. Lurie Children’s Hospital of Chicago, Chicago, IL USA

**Keywords:** Pragmatic trial, Quality improvement, Clinical informatics, Electronic health record, Bronchiolitis

## Abstract

**Background:**

High flow nasal cannula (HFNC) has been increasingly adopted in the past 2 decades as a mode of respiratory support for children hospitalized with bronchiolitis. The growing use of HFNC despite a paucity of high-quality data regarding the therapy’s efficacy has led to concerns about overutilization. We developed an electronic health record (EHR) embedded, quality improvement (QI) oriented clinical trial to determine whether standardized management of HFNC weaning guided by clinical decision support (CDS) results in a reduction in the duration of HFNC compared to usual care for children with bronchiolitis.

**Methods:**

The design and summary of the statistical analysis plan for the REspiratory SupporT for Efficient and cost-Effective Care (REST EEC; “rest easy”) trial are presented. The investigators hypothesize that CDS-coupled, standardized HFNC weaning will reduce the duration of HFNC, the trial’s primary endpoint, for children with bronchiolitis compared to usual care. Data supporting trial design and eventual analyses are collected from the EHR and other real world data sources using existing informatics infrastructure and QI data sources. The trial workflow, including randomization and deployment of the intervention, is embedded within the EHR of a large children’s hospital using existing vendor features. Trial simulations indicate that by assuming a true hazard ratio effect size of 1.27, equivalent to a 6-h reduction in the median duration of HFNC, and enrolling a maximum of 350 children, there will be a > 0.75 probability of declaring superiority (interim analysis posterior probability of intervention effect > 0.99 or final analysis posterior probability of intervention effect > 0.9) and a > 0.85 probability of declaring superiority or the CDS intervention showing promise (final analysis posterior probability of intervention effect > 0.8). Iterative plan-do-study-act cycles are used to monitor the trial and provide targeted education to the workforce.

**Discussion:**

Through incorporation of the trial into usual care workflows, relying on QI tools and resources to support trial conduct, and relying on Bayesian inference to determine whether the intervention is superior to usual care, REST EEC is a learning health system intervention that blends health system operations with active evidence generation to optimize the use of HFNC and associated patient outcomes.

**Trial registration:**

ClinicalTrials.gov NCT05909566. Registered on June 18, 2023.

**Supplementary Information:**

The online version contains supplementary material available at 10.1186/s13063-024-08327-y.

## Background

Bronchiolitis is the most common reason for admission to the hospital in the first year of life and high flow nasal cannula (HFNC) is an increasingly common mode of respiratory support for hospitalized children with bronchiolitis [1]. Between 2012 and 2019, the rate of initiation of HFNC for children presenting to a large children’s hospital emergency department with bronchiolitis significantly increased from 1.3 to 17.0%, without any associated change in the rates of hospital or intensive care unit admission [2]. Between 2010 and 2019, the use of HFNC among children admitted to intensive care at another large children’s hospital significantly increased, while over the same period there was a sevenfold increase in encounters receiving an administrative code for noninvasive ventilation, likely explained by an increase in the use of HFNC [3].

Despite this uptrend in use, data regarding the impact of HFNC on outcomes of children with bronchiolitis are mixed [4]. Randomized, controlled trials have both demonstrated HFNC as reducing the need for escalation of care [5, 6], as well as having no effect on the need for escalation in care [7]. The collective findings of observational studies have been similarly inconclusive, with the use of HFNC associated with decreased [8–11], increased [12], or no difference [13, 14] in the need for intensive care admission or invasive mechanical ventilation. The rise in the use of HFNC for bronchiolitis in the absence of high-quality data regarding efficacy has led to scrutiny regarding the financial costs and personnel time associated with HFNC, while raising the question “is HFNC being overutilized?” [15, 16]. The American Academy of Pediatrics guidelines for the management of bronchiolitis were last updated in 2014 and do not advise on the use of noninvasive respiratory support, underscoring uncertainty regarding how to use HFNC in the management of children with bronchiolitis [17]. In an effort to curb excess HFNC utilization, many children’s hospitals have implemented standard management guidelines, which are increasingly coupled with electronic health record (EHR) workflows, for the use of HFNC for children with bronchiolitis. However, without established best practices for the use of HFNC, it is unclear whether such standardized guidance and related clinical decision support (CDS) meaningfully affect patient outcomes. Moreover, imposing unproven CDS on strained staff risks potentiating burnout in the absence of any benefit to patient care [18].

During the 2022–2023 pediatric respiratory virus surge (“tripledemic”), our own institution experienced an exceptionally high volume of children with respiratory viruses, prompting the development of a local clinical effectiveness guideline outlining a standardized approach to weaning HFNC following its initiation for hospitalized children with bronchiolitis. To promote adherence to the standardized weaning pathway, CDS was designed requiring regular assessments by bedside nurses or respiratory therapists. To ensure that the CDS and related standard weaning pathway is a value-added contribution of patient care, our team designed a randomized, EHR-embedded, pragmatic, Bayesian clinical trial leveraging the support of local quality improvement (QI) teams and processes. We describe the overarching trial design, the embedded electronic workflow, the EHR data informatics pipeline constructed to support trial analyses, the results of trial simulations to inform the trial design, and the statistical analysis plan for the ongoing REspiratory SupporT for Efficient and cost-Effective Care (REST EEC; “rest easy”) trial.

### Aim

The objectives of the REST EEC trial are to (1) determine whether a CDS intervention coupled with a clinical effectiveness guideline is effective at reducing the duration of HFNC for bronchiolitis compared with usual care at a large children’s hospital; (2) demonstrate the utility of embedded randomization and associated EHR data analysis for determining whether CDS is a value-added contribution to patient care; and (3) introduce the concept of “statistically promising,” or a posterior probability of ≥ 0.8, as a meaningful Bayesian statistical conclusion for minimal-risk, QI oriented study questions.

### Study design and setting

REST EEC is a randomized, EHR-embedded, pragmatic, single-center, learning health system trial incorporating QI methodologies. The trial is being conducted at UPMC Children’s Hospital of Pittsburgh (CHP) in the United Sates and is enrolling hospitalized children with bronchiolitis who require HFNC support during admission. CHP is an approximately 300-bed standalone children’s hospital with approximately 25,000 annual inpatient and observation admissions, including between 2000 and 3000 annual admissions to the pediatric intensive care unit (PICU). The PICU has 36 dedicated beds but routinely exceeds capacity during the winter and respiratory virus season months, reaching daily censuses as high as 60 during peak patient volumes. CHP policies require children receiving HFNC to be cared for in an intensive care unit and all children with bronchiolitis who are greater than 1 month old, newly admitted to the hospital (not already admitted to the neonatal or cardiac intensive care units) and without unrepaired, cyanotic heart disease are cared for by the PICU service.

CHP uses the Cerner Millennium EHR (Oracle Cerner Corporation, Kansas City, MO). A CHP enterprise data warehouse (EDW) relies on an Oracle Data Warehouse solution (Oracle Corporation, Austin, TX), Informatica ETL (extract, transform, and load) software (Redwood City, CA) for movement and transformation of data from local EHR data sources, and Qlik software (Qlik, King of Prussia, PA) for up-to-date data visualization to support QI initiatives. This description of a clinical trial was developed to follow the SPIRIT reporting guidelines (Supplemental Checklist).

### Data collection processes

The design and statistical analysis plan for REST EEC were informed by observational data from the EHR. To inform the Bayesian adaptive design, trial simulations were performed based on current data on bronchiolitis admissions from recent years at CHP. An Informatica ETL pipeline was developed to harvest the initial data to support simulations, with the intent of then leveraging the same pipeline to both monitor trial progress and support ensuing analyses. The CHP EDW has a low latency refresh, harboring data that are commonly only seconds behind the production EHR used by clinicians for patient care. To facilitate data retrieval, a subset of the warehouse contains a “staged” rolling 3–4 years of curated, transformed, and cleansed EHR data that are highly indexed to optimize query times. The Informatica pipeline queries this staged environment and produces a single table output that contains salient patient demographics, covariates of interest, and outcomes data. Queries can be run ad hoc, and the pipeline is also being attached to a Qlik dashboard interface that refreshes nightly and will provide data on enrollment, including total enrolled and summary statistics of treatment assignments. The curated REST EEC data table can be linked with an existing intensive care EDW data mart that automatically refreshes nightly and contains salient critical care data elements such as duration of mechanical ventilation, as well as pediatric risk of mortality IV score, and pediatric logistic organ dysfunction 2 scores, as previously described, to support interim and final trial analyses [19]. As data to support trial analyses are harvested from clinical documentation, data quality is reliant on best patient care practices and periodic external audits from accrediting bodies such as the Joint Commission. Missing data will be reported as such and outliers will be adjudicated by a nurse informaticist member of the study team.

Trial conduct, interim analyses, and final analyses are also relying on observational data sources, including both the EHR and local data submitted to the Pediatric Health Information Systems (PHIS) database, which is a large, multicenter, benchmarking database for children’s hospitals that harbors predominantly administrative data including encounter dates, care-related charges, and diagnosis codes. PHIS offers additional data elements not readily retrievable from the EDW, including geographically adjusted costs of care, complex chronic comorbidities flags as defined by Feudtner et al. [20], and an additional set of risk scores providing predicted mortality (based on administrative admission data) and expected mortality (based on administrative data from the entire encounter) for use as additional balance measures. PHIS is maintained by Children’s Hospital Association (Lenexa, KS) and can be accessed using a web application version of SAP Business Objects (SAP, Walldorf, Germany). PHIS data are deidentified but can be linked to local, single center patient data via a decryption key possessed by designated individuals at the site, as we have previously done for observational studies. A data monitoring committee was not deemed necessary as the intervention is minimal risk.

### Guideline eligibility criteria and patient inclusion plan

**Fig. 1:**
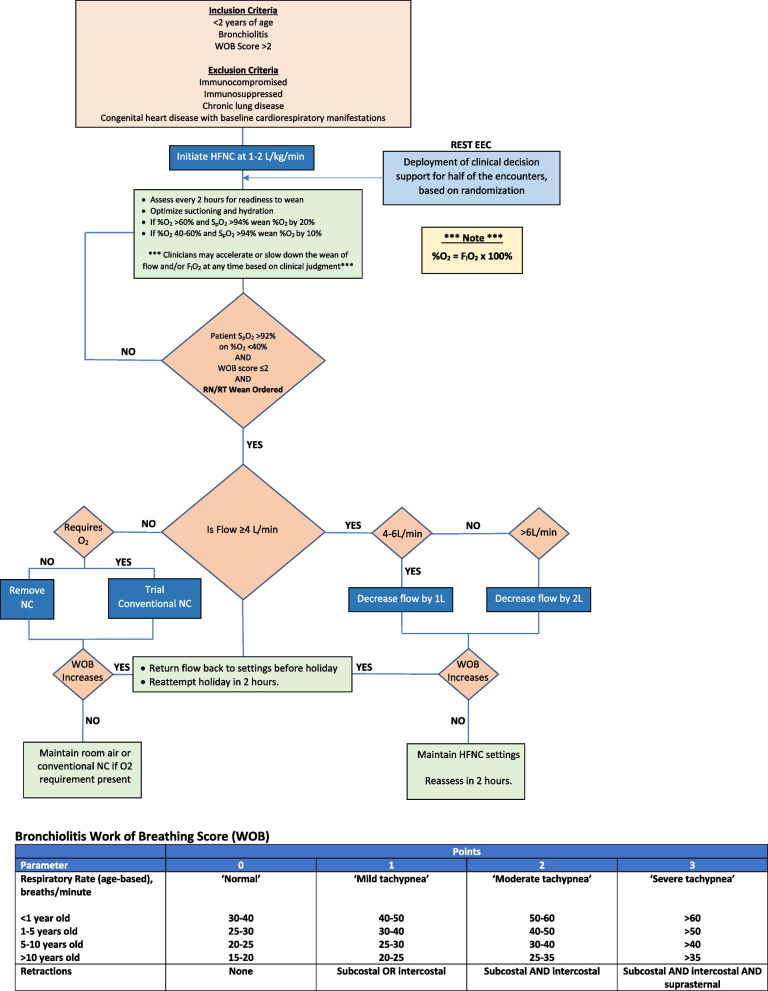
The standardized, high flow nasal cannula weaning pathway including eligibility criteria (light orange box), weaning criteria (dark orange boxes), the point of deployment of the REST EEC randomized workflow (light blue box), specific management guidance (dark blue boxes), ongoing management guidance (green boxes), and the work of breathing score used to guide objective patient assessments (table below the algorithm)

Clinicians are guided to include patients in the REST EEC trial if the following criteria are met (Fig. 1): (1) a diagnosis of bronchiolitis per the clinical team; (2) less than 2 years of age; (3) a work of breathing (WOB) score > 2 (score calculation displayed in Fig. 1). Because a dysfunctional immune response can result in an atypical and erratic disease course, and because both chronic lung disease and certain congenital heart diseases can sufficiently alter baseline work of breathing in a way not accommodated by the WOB score, guidance is provided to clinicians to exclude patients from the REST EEC trial if any of the following criteria are present: (1) immunocompromised or immunosuppressed status; (2) presence of chronic lung disease; (3) presence of congenital heart disease with cardiorespiratory manifestations. As the REST EEC trial relies on clinicians for assessment of these eligibility criteria, patients will ultimately be included in the primary analysis if clinicians place the orders required for randomization and study entry, the patient age is < 2 years, and the patient encounter is associated with a primary or secondary diagnosis code compatible with a viral lower respiratory tract infection.

### Randomization

The intervention is the provision of clinical decision support to guide bedside nurses and respiratory therapists in the systematic weaning of HFNC for children with bronchiolitis. The control is usual care, which consists of a clinical pathway for HFNC weaning without associated clinical decision support. Ordering clinicians are the target of randomization, but the unit of randomization is a clinician-patient decision moment. Accordingly, each time a clinician has decided to initiate HFNC for a patient eligible for REST EEC, randomization will occur in an unblinded, automated, and 1:1 ratio between the intervention and usual care arms. Only the CDS intervention is randomized and all other components of care are not specified by the study design, instead proceeding according to usual care. As the intervention involves CDS to reinforce an existing standard of care and the endpoint is duration of HFNC, a respiratory support modality that is the focus of de-implementation campaigns and not considered an established standard of care, the University of Pittsburgh Institutional Review Board approved waiver of consent for the REST EEC trial. Randomization is automated and cued by placement of a “HFNC for Bronchiolitis” Cerner PowerPlan (orderset) and based on whether a specific digit in the encounter identifier, which is a Cerner Millennium database key not readily apparent to clinicians and specific to a patient hospital encounter, is even or odd. Encounter identifiers are assigned sequentially as patients are registered for an interaction with the healthcare system, ensuring that the assignments are equally and randomly distributed between the 2 trial arms.

### Current clinical operations prior to study initiation

Figure 1 is the HFNC weaning clinical effectiveness guideline available to clinicians at CHP at the time of REST EEC initiation. HFNC is provided from children < 2 years old using the either the Fisher & Paykel Optiflow circuit or the Airvo 2 heated high flow device with an Airvo circuit and Optiflow junior cannulas (all manufactured by Fisher & Paykel Healthcare, Auckland, New Zealand) and initiated at 1–2 mL/kg of flow per clinician discretion and as indicated in the clinical effectiveness guideline. HFNC management is overseen by board-certified or eligible pediatric intensivists and duration is determined on an individual basis according to patient response and the need for escalation versus weaning. Following development of the guideline, an education session was held with all respiratory therapists detailing the guideline contents and responding to questions. Pediatric clinical effectiveness guidelines are available for all UPMC clinicians on the health system’s intranet and the HFNC weaning guideline was first made available in the winter of 2022–2023. A laminated copy of the guideline is also placed on the HFNC equipment and therefore available in hard copy at the bedside of each patient receiving HFNC. The guideline is revisited at least annually by a committee of intensivists, respiratory therapists, nurses, and informaticians with updates made as needed during those annual reviews. Prior to REST EEC, HFNC was initiated after placement of an “Oxygen Therapy” Cerner PowerPlan in which clinicians could select between 2 options: (1) “Do Not Wean Oxygen Without Physician Order/Modification” (*sic*); (2) “RT/RN may wean O2, following the weaning order” (*sic*). The choice of HFNC as the delivery device (as opposed to, for example, a Venturi mask) was specified within the “Oxygen Therapy” PowerPlan. Among the patients receiving HFNC for bronchiolitis in the PICU whose data were used for trial simulations, none received an order for standardized weaning prior to the deployment of the REST EEC study.

### Study intervention and associated workflow

A new electronic workflow was developed to deploy the REST EEC trial. Clinicians, including pediatric intensivists, pediatric intensive care fellows, pediatric emergency medicine physicians, general pediatrics residents, emergency medicine residents, emergency and intensive care nurse practitioners, and emergency and intensive care physician assistants, are responsible for diagnosing bronchiolitis in children. The intervention arm consists of clinical decision support that includes an order bearing instructions for the HFNC weaning process, as outlined in the guidelines, that is placed in the “Respiratory Care” section of the Cerner orders. This order is also linked to a scheduled task that appears on both the nursing and respiratory therapy tasks lists, requesting that a WOB score be documented every 2 h and the ability to wean HFNC assessed by either of those clinicians caring for the patient. This intervention differs from the usual care arm, in which the HFNC weaning guideline is accessible to the clinical team via the UPMC intranet, but decisions to wean HFNC are made ad hoc by the clinical team. By requiring every 2-h assessments, the investigators hypothesize that the duration of HFNC will be shorter among patients randomized to the intervention arm of REST EEC, as compared to the usual care arm. The REST EEC design does not include any guidance on therapies outside the use of HFNC. The decision to discontinue or modify HFNC is left to the bedside clinician responsible for patient care; the CDS serves as a guide.

**Fig. 2:**
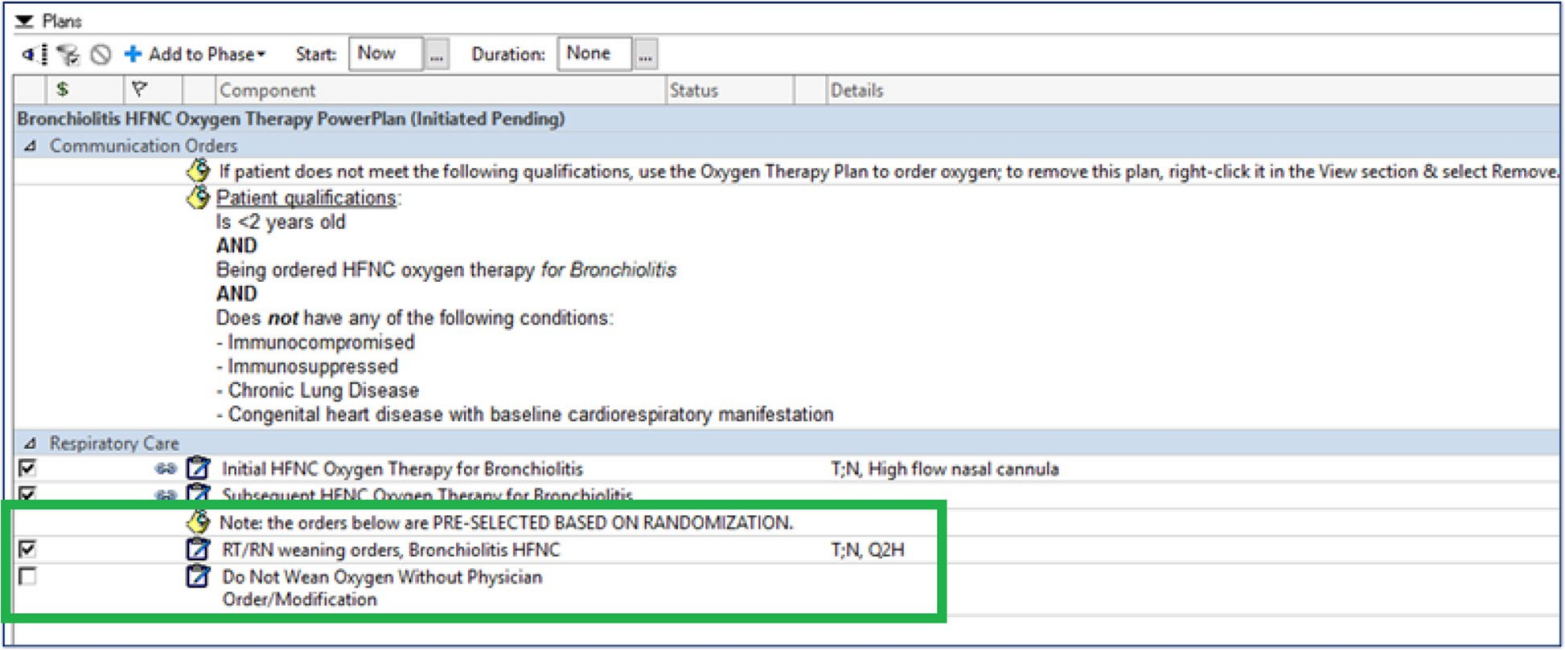
Screenshot of the Cerner “Bronchiolitis HFNC Oxygen Therapy PowerPlan.” Eligibility criteria are listed at the top of the PowerPlan, with instructions to clinicians to remove the PowerPlan if the patient does not meet criteria. The green box outlines the section of the PowerPlan that is pre-selected according to the patient’s randomized assignment

To accommodate the unique patient population of interest, the clinical effectiveness guideline workflow, and the need for randomization, a “HFNC for Bronchiolitis” PowerPlan was constructed leveraging features of Cerner’s PowerFlex PowerPlan technology. PowerFlex PowerPlans allow for dynamic PowerPlan characteristics according to programmed rules that can draw upon individual patient characteristics. To ensure that patients > 2 years of age are not randomized, the “HFNC for Bronchiolitis” PowerFlex PowerPlan cannot be ordered for patients 2 years or older. If attempting to place the PowerPlan, clinicians receive a message stating that the patient is not eligible for the order and asking them to remove the PowerPlan. For patients < 2 years old, the REST EEC trial eligibility criteria are displayed at the top of the PowerPlan and clinicians are instructed to sign the PowerPlan only if patients meet the criteria (Fig. 2). The REST EEC study arms, one for the intervention and one for usual care, are both available as options within the PowerPlan, alongside instructions to clinicians stating “The pre-selected orders below are based on randomization and should only be changed if the physician/APP thinks it is in the best interest of the patient.” The randomized assignment is pre-selected using the PowerFlex feature when the PowerPlan is opened but can be changed if a clinician has a strong preference. The intervention group are those patients for whom CDS is ordered and the control group are those patients who receive usual care without CDS. A new WOB score documentation field was also built in a segment of the electronic flowsheet that overlaps with nursing and respiratory therapist workflows, allowing both disciplines to document the score as requested by the HFNC weaning guideline. REST EEC is deployed as part of usual care and all ancillary and post-trial care occur according to usual care.

### Endpoints

The primary outcome is duration of HFNC measured from the time of initiation of the HFNC PowerPlan to final weaning off of HFNC support evidenced by documentation of nasal cannula flow less than 4 L per minute among patients who do not require noninvasive or invasive ventilation following randomization, up to 30 days. The design is open label with only outcome assessors being blinded so unblinding will not occur. For patients requiring noninvasive or invasive ventilation following randomization, the duration of HFNC will be measured following last documented mode of positive pressure ventilation preceding placement on HFNC. Secondary outcomes include respiratory support free days, organ support free days, PICU length of stay, hospital length of stay, time to oral intake, respiratory distress measured by the WOB score, geographically adjusted cost of care for hospitalization, and mortality. Data to calculate the endpoints will be collected from the EHR and the PHIS database. Follow-up is limited to the hospitalization during which the patient received HFNC and long-term follow-up beyond hospitalization is not planned for the REST EEC trial.

### Statistical model, simulation results, and statistical analysis plan

The primary endpoint analysis will be conducted according to each patient’s randomized assignment. To provide an estimate of the effect of the intervention on the primary outcome of duration of HFNC, we use a semiparametric Bayesian proportional hazards regression model. This model has two components. The baseline hazard component models the underlying chance of weaning off of HFNC over time for standard of care. The hazard ratio (HR) component models the CDS intervention effect as the proportional change in the chance of weaning for those randomized to CDS. Because the exact form of the baseline hazard is unknown, we specify a flexible model which can accommodate a wide range of possibilities. Specifically, the baseline hazard is modeled using cubic M-splines with two boundaries and two internal knots at equally spaced percentiles of the distribution of uncensored HFNC durations with a flat Dirichlet prior distribution over the spline coefficients. A weakly informative N(0, 2.5) prior is used for the intervention log(HR) parameter. This prior is centered at a HR of 1 implying no difference between standard of care and CDS and has a negligible impact on the posterior treatment effect estimate. The model is implemented using the stan_surv() function in the rstanarm modeling package [21].

**Fig. 3:**
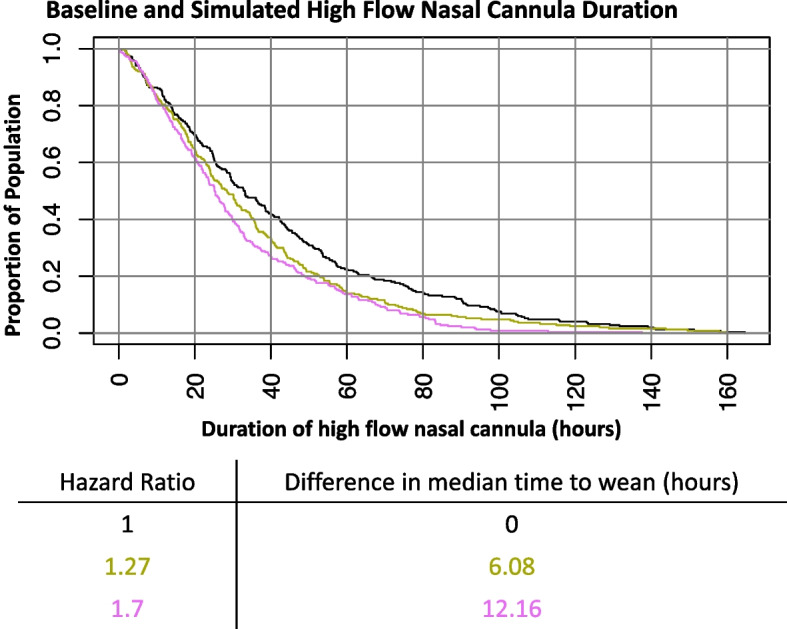
The baseline population duration of high flow nasal cannula (HFNC) and simulated duration with an intervention effect size of a median duration 6.08 h less than baseline (hazard ratio [HR] 1.27; gold line and numbers in table) and with an intervention effect size of a median duration 1.7 h less than baseline (hazard ratio [HR] 1.7; pink line and numbers in table)

**Fig. 4:**
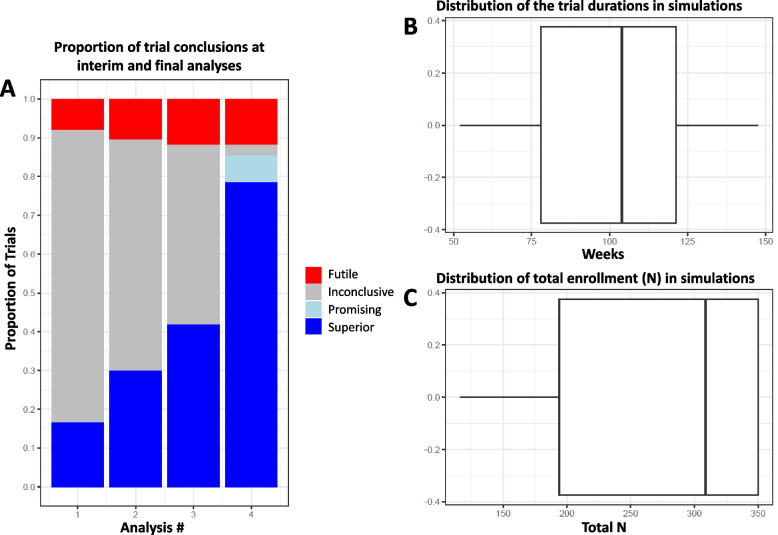
Results of trial simulations: **A** the proportion of trial conclusions at 3 interim analyses and 1 final analysis deemed futile (red), inconclusive (gray), intervention promising (light blue), and intervention superior (dark blue). Trial simulations indicate that by assuming a true hazard ratio effect size of 1.27, equivalent to a 6-h reduction in the median duration of HHFNC, and enrolling a maximum of 350 children, there will be a > 0.75 probability of declaring superiority (interim analysis posterior probability > 0.99 or final analysis posterior probability > 0.9) and a > 0.85 probability of declaring superiority or the CDS intervention showing promise (posterior probability of > 0.8); **B** distribution of total duration of the trials in simulations; **C** distribution of total enrollment in simulations

As REST EEC uses an adaptive Bayesian design, virtual trial simulations were conducted to understand statistical design operating characteristics such as sample size, trial duration, and power for putative trial scenarios with a given intervention effect size and to determine feasibility of the planned design. To inform the Bayesian analysis plan, data were extracted from the CHP EDW staged environment, which contained all CHP encounters for the preceding 3 years. Supplemental Table 1 displays the data dictionary used to define the preliminary data to inform simulations. There were 549 encounters that received HFNC with a primary or secondary diagnosis of bronchiolitis. The estimated median time to weaning was 35 h (Fig. 3). Several trial scenarios were explored, with varied accrual rates and effect sizes as measured by the difference in median time to weaning between the CDS intervention arm versus usual care. For each scenario, 5000 virtual trials were simulated and the behavior of each trial was tracked to determine the design operating characteristics. An anticipated effect evidenced by a HR of 1.27, or a 6-h difference between the median time to weaning in the CDS versus usual care arm, was selected as providing a clinically significant but realistic effect of the intervention. HFNC weaning in the CHP PICU commonly occurs following PICU morning rounds. A 6-h decrease in HFNC duration therefore commonly translates into a morning rather than afternoon discharge out of the PICU, which can subsequently facilitate earlier hospital discharge. Three interim analyses (at 12 months, 18 months, and 24 months after study initiation) and one final analysis (at the maximum sample size) will be performed. At each interim analysis, the study will declare superiority of CDS if the posterior probability of HR > 1 exceeds 0.99 or futility of CDS if the posterior probability of HR < 1.15 exceeds 0.8, meaning that futility will be declared if the probability is > 0.8 that the effect of clinical decision support is less than a 4-h reduction in HFNC duration. If neither condition is met, the study will continue to the next interim or final analysis. At the final analysis, CDS will be declared superior if the posterior probability of HR > 1 exceeds 0.9. A clinically actionable posterior probability threshold at which the CDS would be considered “statistically promising” and left in place following trial conclusion was determined to be 0.8 after conferring among the investigators and clinical leadership. Planning for balanced randomization between the CDS intervention and usual care, with a maximum sample size of 350 over approximately 2–3 years (100–150 patients per year), trial simulations indicate there will be a > 0.75 probability of declaring superiority (either interim analysis posterior probability of HR > 1 greater than 0.99 or final analysis posterior probability of HR > 1 greater than 0.9) and a > 0.85 probability of declaring superiority or the CDS intervention showing promise (final analysis posterior probability of HR > 1 greater than 0.8) (Fig. 4). This design was deemed feasible as approximately 150 children with bronchiolitis < 2 years old receive HFNC annually at the study site. Planned secondary analyses include evaluating the primary endpoint among select subgroups, including children treated with beta agonists therapy, children treated for pneumonia, children with complex chronic conditions, children receiving positive pressure ventilation, and children co-diagnosed with croup. The electronic data collection strategy has been designed to encode missing data as absent, avoiding the need for imputation. The primary analysis will be intention to treat (ITT) and a secondary, modified ITT analysis will be performed based on assignments that may have been altered by the ordering clinicians.

### Monitoring and ensuring compliance

**Fig. 5:**
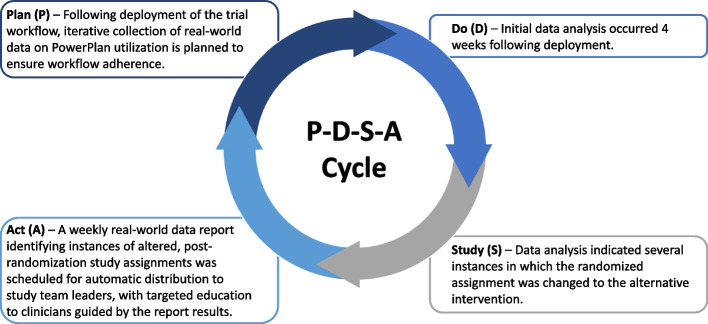
Plan-do-study-act cycle example that demonstrates how quality improvement methodology is used to support ongoing conduct of the trial

**Fig. 6:**
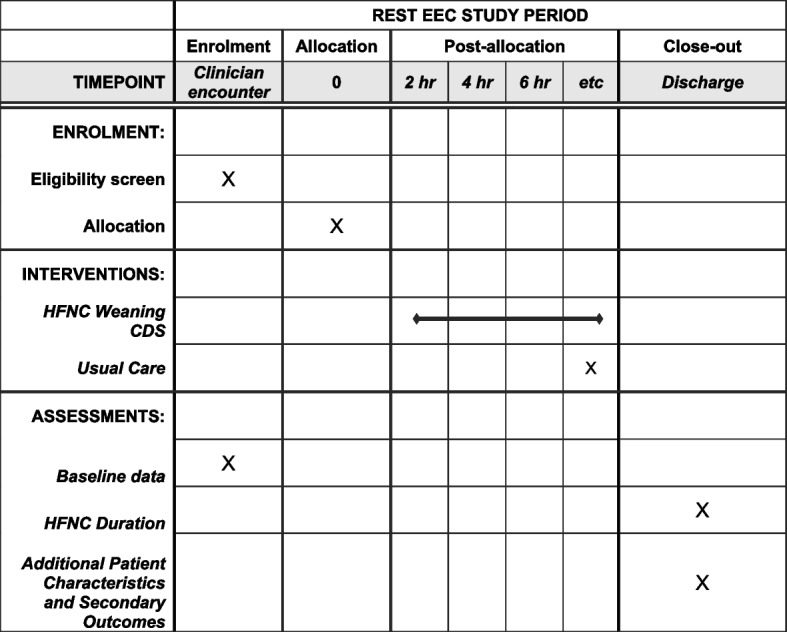
Schedule of enrolment, interventions, and assessments

REST EEC is a pragmatic trial embedded within real world workflows and relies on minimal infrastructure components that would be typical in a traditional trial, such as research coordinators tracking compliance with an assigned study intervention. Trial oversight and the steering committee consist of members of relevant hospital operations teams. This includes clinical intensivist leadership, physician informaticians, nurse informaticians, and nurse improvement specialists. Summary enrollment data are emailed and reviewed weekly by a physician informatician and 2 nurse informaticians. To ensure adoption of the “HFNC for Bronchiolitis” order and ongoing adherence to the study workflow, the trial relies on CHP QI resources to support its ongoing conduct. Nurse clinician educators and physician faculty clinical leaders in the emergency department and PICU informed their respective workforces about the trial design and expectations for frontline clinicians. As QI methods emphasize the importance of patient engagement and clinician feedback, the REST EEC trial was integrated into existing patient and clinician QI structures at the study site. Upon admission, a bedside nurse educates all patient caregivers on the caregivers’ ability to advocate and intervene in patient care. This includes questioning care team decisions, including the decision to wean HFNC, as well as calling a “Condition Help” if a parent feels unheard about a patient safety concern. The trial has been iteratively reviewed in PICU physician quality reporting system (PQRS) sessions that serve as the main forum for introducing and updating the PICU physician faculty on the progress of QI initiatives. Biannual updates on trial progress are planned for PQRS sessions, during which time an open forum for discussion and feedback from physicians, nurses, respiratory therapists, pharmacists, and social workers will occur. The PICU QI forum, an in-hospital safety event reporting system, as well as ad hoc communication to PICU clinical leadership, will be used to collect reports of unintended effects of trial interventions or conduct. Assessment and management of any reports of unintended, adverse effects will be handled by the PICU clinical leadership in communication with the REST EEC study team. The data collection strategies are the same used to track progress of other inpatient CHP performance improvement initiatives and are performed by a quality improvement data team at UPMC. This team includes nurse informaticians, a physician informatician who also serves as the study principal investigator, a data analyst, a project manager, and a nurse quality improvement specialist. Iterative plan-do-study-act (PDSA) cycles are used to monitor the trial and provide targeted education to the workforce (Fig. 5). “Study” events within the PDSA cycle will occur following the planned interim analyses and as needed based on ad hoc feedback mechanisms that constitute the trial’s QI processes. In addition to declaring superiority, futility, or the need to continue the trial, the PDSA cycles will also focus on documentation of WOB scores and make a determination regarding whether additional education sessions are needed for nurses and respiratory therapists if documentation is infrequent. Figure 6 displays the schedule of trial activities. Protocol changes will be filed with the study institutional review board and the trial registry information will be updated.

### Dissemination plan

Two manuscripts are planned for the REST EEC trial: (1) this initial description of the study design and statistical analysis plan; (2) reporting the results of the trial. Authorship of the manuscripts will be determined according to guidelines established by the International Committee of Medical Journal Editors [22].

### Trial status

The trial is registered at ClinicalTrials.gov as NCT05909566. The present manuscript describes information contained in the REST EEC Protocol and Statistical Analysis Plan version 1.0, dated December 6, 2023. The “HFNC for Bronchiolitis” PowerPlan went live in CHP Cerner on December 12, 2023. As of January 9, 2024, the PowerPlan has been used 51 times, or an enrollment rate of 1.8/day. Recruitment is expected to continue until approximately January 1, 2026.

## Discussion

There are increasing concerns that the use of HFNC for the management of bronchiolitis in the United States of America is suboptimal, contributing to excess costs and amplifying strains of both personnel and material resources at children’s hospitals during surges of patients afflicted with respiratory viruses [23]. In this context, any effort to safely reduce the utilization of this resource-intensive therapy will be beneficial and will align well with patient-centered outcomes. The REST EEC trial will examine whether CDS supporting standardized HFNC weaning will result in a shorter duration of HFNC among children with bronchiolitis compared to usual care. By randomizing at the level of order placement, as opposed to a quasi-experimental before-after design, the REST EEC trial will more accurately assess for a potential causal relationship between an EHR-embedded HFNC weaning workflow and the duration of HFNC.

As both a demonstration and potential solution to overutilization, single and multicenter de-implementation campaigns have recently worked to reduce the use of HFNC specifically for bronchiolitis, without any reported negative impact on patient care [16, 24, 25]. Importantly, calls for HFNC de-implementation have commonly cited the lack of efficacy data for improving outcomes such as duration of hospitalization or intensive care admission. Constructive opposition has noted that the absence of conclusive data regarding the efficacy of HFNC does not constitute evidence of the magnitude of overutilization of HFNC [26]. Additional data are needed to better understand the impact of HFNC on patient dyspnea, air hunger, agitation, and work of breathing, all of which may be unrelated to the duration of illness or the need for escalation of respiratory support to invasive or noninvasive ventilation. Accordingly, REST EEC examines a potential strategy for more efficient utilization of HFNC while leaving the decision to initiate the therapy to bedside clinicians.

Integrating REST EEC into usual care workflows allows the trial to capitalize on existing care infrastructure, resulting in a more cost-effective deployment. Embedded randomization is a common tactic to optimize software applications in non-healthcare industries but is not yet widely applied in medicine to refine EHR workflows [27]. The insights generated from REST EEC will be rapidly translated into practice, with CDS remaining in place for all children with bronchiolitis if the intervention proves statistically promising compared to usual care and is deemed to be useful by clinicians following review of the final analyses, and the CDS being turned off if there is an insignificant difference in HFNC duration between the intervention and usual care arms. De-implementing ineffective CDS is important for reducing click burdens on frontline staff and mitigating the risk of EHR-associated burnout. REST EEC’s novel, Bayesian design introduces the concept of a posterior probability > 0.8 representing a sufficient threshold to declare a minimal risk intervention superior to the control arm. This full cycle application of Bayesian analysis aligns with the original vision of “evidence-based medicine” described by Dr David Eddy, who coined the term while noting the need for more objective, probabilistic decision-making by physicians that relied on Bayes theorem [28].

HFNC has emerged as a common modality for pediatric respiratory support over the past 2 decades [29]. Its ease of use and noninvasive nature have fostered its adoption as a form of respiratory support; however, the use of HFNC is more resource intensive than other noninvasive methods of respiratory support such as facemasks or low-flow nasal cannula, commonly requiring the expertise of certified respiratory therapists to setup and titrate in the United States of America. REST EEC will help to answer whether standardized HFNC weaning coupled with CDS will result in a shorter duration of HFNC compared to usual care among children with bronchiolitis. Through incorporation of the trial into usual care workflows, relying on QI tools and resources to support trial conduct, and relying on Bayesian inference to determine whether the intervention is superior to usual care, REST EEC is emblematic of a learning health system intervention that blends health system operations with active evidence generation [30].

### Supplementary Information

Additional file 1: SPIRIT checklist

Additional file 2: Supplemental Table 1 Data dictionary

## Data Availability

Data and materials are the property of UPMC and the University of Pittsburgh. There is no individual clinical trial participant-level data sharing plan; access is granted for approved use cases only.

## Abbreviations

CDS: Clinical decision support
CHP: UPMC Children’s Hospital of Pittsburgh
EDW: Enterprise data warehouse
EHR: Electronic health record
ETL: Extract, transform, and load
HFNC: High flow nasal cannula
HR: Hazard ratio
PDSA: Plan-do-study-act
PHIS: Pediatric health information systems
PICU: Pediatric intensive care unit
QI: Quality improvement
REST EEC: REspiratory SupporT for Efficient and cost-Effective Care
WOB: Work of breathing
