# Supplementary material for: A randomized, embedded, pragmatic, Bayesian clinical trial examining clinical decision support for high flow nasal cannula management in children with bronchiolitis: design and statistical analysis plan
Source: Trials. 2024 Jul 16;25:484. doi: 10.1186/s13063-024-08327-y (PMC11253479; doi:10.1186/s13063-024-08327-y)
Supplement: Supplementary file 2 — Additional file 2: Supplemental Table 1 Data dictionary [file 13063_2024_8327_MOESM2_ESM.docx]

**Supplemental Table 1.** Data dictionary.

| **Variable** | **Definition** | **Logic** |
| --- | --- | --- |
| EncounterID | Unique identifier for hospital encounter | Safe harbor deidentified for data transfer |
| Person ID | Unique identifier for patient | Safe harbor deidentified for data transfer |
| AdmitWeek | Week of admission | Coded as 1 (first week of January) through 52 (last week of December) |
| Age | Age on day of admission |  |
| Sex | Sex recorded in electronic record at time of admission |  |
| Race | Race recorded in electronic record at time of admission |  |
| Asthma | Any ICD-10 asthma code for status asthmaticus, asthma exacerbation or reactive airway disease AND >1 treatment with albuterol AND treatment with systemic corticosteroids | Y/N Include diagnosis code, description and type; Include ALL diagnosis types (final and secondary) Codes: J45.20; J45.21; J45.22; J45.30; J45.31; J45.32; J45.40; J45.41; J45.42; J45.50; J45.51; J45.52; J45.901; J45.902; J45.909; J45.990; J45.991; J45.998 |
| Bronchiolitis | Any diagnosis of bronchiolitis | Y/N Include diagnosis code, description and type; Include all diagnosis types (final and secondary) Codes: J21.0; J21.1; J21.8; J21.9; J84.115; B97.4; J12.1 |
| Pneumonia | Any diagnosis ICD-10 pneumonia code AND treatment with IV antibiotics for pnuemonia or sepsis | Y/N Patient needs to have both a diagnosis of pneumonia (as a final or secondary code) and one of the antibiotics Codes: A37.00; A37.01; A37.10; A37.80; A37.81; A37.90; A37.91; A40.3; B95.3; B96.0; B96.1; J09.X1; J10.00; J10.01; J10.08; J11.00; J11.08; J12.0; J12.1; J12.2; J12.3; J12.81; J12.82; J12.82; J12.89; J12.9; J13; J14; J15.0; J15.1; J15.20; J15.211; J15.212; J15.29; J15.3; J15.4; J15.5; J15.6; J15.7; J15.8; J15.9; J16.0; J16.8; J17; J18.0; J18.1; J18.8; J18.9; J20.0; J82.81; J84.111; J84.116; J85.1; J85.2; J95.851; P23.0; P23.2; P23.6; P23.8; P23.9; Z87.01  Intravenous antibiotics: ampicillin; ampicillin-sulbactam; cefepime; cefixime; cefotaxime; cefoxitin; ceftazidime; ceftriaxone; clindamycin; ciprofloxacin; levofloxacin; meropenem; piperacillin-tazobactam; tobramycin; vancomycin; avibactam-ceftazidime |
| InvasiveMechanicalVentilation | The patient received invasive mechanical ventilation via an endotracheal tube | Y/N if they were intubated during their stay |
| NoninvasiveMechanicalVentilation | The patient received noninvasive mechanical ventilation | Y/N if they had non-invasive mechanical ventilation during their stay |
| Croup | Any diagnosis of croup | Y/N Include diagnosis code, description and type; Include all diagnosis types Code: J05.0 |
| ShockPrior | Received an infusion (2 or more documented hours) of epinephrine, norepinephrine, or dopamine prior to or at the time of HFNC | Y/N Patient received one of the following medications within 1 hour before or any time during HFNC; norepinephrine; milrinone; epinephrine; dopamine; vasopressin; dobutamine |
| DurationHFNC | Duration of HFNC treatment | Difference between the first and last timestamp a patient is documented as having HFNC in hours; for patients who receive either invasive or noninvasive ventilation, the first HFNC timestamp is the first hat follows the last documented invasive or noninvasive ventilation support |
| ICULOS | Number of ICU days for the encounter | Number of ICU Calendar days |
| HospLOS | Number of hospital days for the encounter | Difference between the discharge date and the admission date of the encounter, in days |
| TimeToPO | Number of hours between randomization and first documented oral intake | Randomization time to the first documented oral intake |
| DurationSuppOxygen | Number of hours between first and last documentation of any form of supplemental oxygen | Oxygen therapy equal to anything other than 'room air' |
| AreaDeprivationIndex | Area deprivation index mapped to census block |  |
| ChildOpportunityIndex | Child opportunity index mapped based on zip code in the PHIS database |  |
| ComplexChronicComorbidity | Presence of a complex, chronic comorbidity (CCC), as defined by PHIS |  |
| Cardiovascular | CCC |  |
| Gastrointestinal | CCC |  |
| HematologicImmunologic | CCC |  |
| Malignancy | CCC |  |
| Mental Health Disorder | CCC |  |
| Metabolic | CCC |  |
| Neurologic and Neuromuscular | CCC |  |
| NICU | CCC |  |
| OtherCongenitalorGeneticDefect | CCC |  |
| PrematureandNeonatal | CCC |  |
| RenalandUrologic | CCC |  |
| Respiratory | CCC |  |
| SecondaryDxMentalHealthDisorder | CCC |  |
| TechnologyDependent | CCC |  |
| TPN | CCC |  |
| Transplant | CCC |  |
| ICU | CCC |  |
| EncounterCost | Cost of encounter, geographically adjusted, from PHIS |  |
| **Abbreviations:** CCC, complex chronic comorbidity; HFNC, high flow nasal cannula; ICD-10, international classification of diagnoses version 10;  ICU, intensive care unit; LOS, length of stay | | |
